# The impact of cholesterol deposits on the fibrillar architecture of the Achilles tendon in a rabbit model of hypercholesterolemia

**DOI:** 10.1186/s13018-019-1217-7

**Published:** 2019-06-10

**Authors:** Andrzej Steplewski, Jolanta Fertala, Ryan Tomlinson, Kevth’er Hoxha, Lin Han, Ocean Thakar, Jason Klein, Joseph Abboud, Andrzej Fertala

**Affiliations:** 1 Department of Orthopaedic Surgery, Sidney Kimmel Medical College, Thomas Jefferson University, Curtis Building, Room 501, 1015 Walnut Street, Philadelphia, PA 19107 USA; 2 School of Biomedical Engineering, Science & Health Systems, Drexel University, Philadelphia, PA USA; 3 Rothman Institute of Orthopaedics, Thomas Jefferson University, Philadelphia, PA USA

**Keywords:** Hypercholesterolemia, Tendon, Collagen fibrils

## Abstract

**Background:**

Increased tendon pain and tendon damage is a significant complication related to hyperlipidemia. Unlike the well-established pathogenesis associated with increased serum concentrations of total cholesterol, triglycerides, and low-density lipoprotein in atherosclerotic cardiovascular disease, the role of hyperlipidemia in promoting tendon damage remains controversial and requires mechanistic clarity.

**Methods:**

In this study, we analyzed the consequences of hypercholesterolemia on the integrity of the collagen-based architecture of the Achilles tendon. The Achilles tendons from rabbits fed with normal-cholesterol (nCH) and high-cholesterol (hCH) diets were analyzed. We studied the morphology of tendons, distribution of lipids within their collagen-rich milieu, the relative amounts of fibrillar collagen I and collagen III, and selected biomechanical parameters of the tendons at the macroscale and the nanoscale.

**Results:**

Histological assays of hCH tendons and tenosynovium demonstrated hypercellular areas with increased numbers of macrophages infiltrating the tendon structure as compared to the nCH tendons. While Oil Red staining revealed lipid-rich deposits in the hCH tendons, hybridization of tendon tissue with the collagen hybridizing peptide (CHP) demonstrated damage to the collagen fibers. Fourier-transform infrared (FTIR) spectra showed the presence of distinct peaks consistent with the presence of cholesterol ester. Additionally, the hCH tendons displayed regions of poor collagen content that overlapped with lipid-rich regions. The hCH tendons had a substantial fourfold increase in the collage III to collagen I ratio as compared to the nCH tendons. Tendons from the hCH rabbits showed poor biomechanical characteristics in comparison with control. The biomechanical changes were evident at the macrolevel and the nanolevel of tendon structure.

**Conclusions:**

Our findings support the hypothesis that hypercholesterolemia coincides with the weakening of the tendons. It is likely that the intimate contact between collagen fibrils and cholesterol deposits contributes to the weakening of the fibrillar structure of the tendons.

## Background

Increased tendon pain and tendon damage are clinically significant potential complications related to hyperlipidemia [1–3]. Unlike the well-established pathogenesis associated with increased serum concentrations of total cholesterol (TC), triglycerides (TG), and low-density lipoprotein (LDL) in atherosclerotic cardiovascular disease, the role of hyperlipidemia in promoting tendon damage remains controversial and requires mechanistic clarity.

Studies in humans have indicated a possible association between hyperlipidemia and increased risk for tendon damage. For example, Mathiak et al. have found that 34 of 41 (83%) patients with Achilles tendon ruptures had elevated serum cholesterol concentration [4]. Researchers also demonstrated a positive relationship between the damage of the Achilles tendon and familial hyperlipidemia [1]. Another report documented musculoskeletal system manifestations among 38% of patients with juvenile familial hyperlipidemia; administering lipid-lowering drugs improved the pathological manifestations in 63% of these patients [5]. Injuries of the rotator cuff may also be linked to hyperlipidemia based on findings that, among the approximately 23% of individuals older than 50 with a rotator cuff tear, serum concentrations of TC, TG, and LDL were significantly higher than in patients over 50 years of age without a rotator cuff tear [2]. Similar studies by other researchers, however, demonstrated no clear relationship between the serum concentrations of TC and TG and the occurrence of rotator cuff tears [6].

Meanwhile, studies in animal models have demonstrated a link between hyperlipidemia and significant alterations of the mechanical properties of tendons [2, 7–10]. In one example, assays of the supraspinatus tendons from hypercholesterolemic mice, rats, and monkeys demonstrated consistently increased tendon stiffness and elastic modulus compared to corresponding parameters of control tendons [11]. Other studies have revealed adverse changes in rats fed with high-cholesterol diet; among these, one study highlighted a significant reduction of normalized stiffness of the tendons in hypercholesterolemic rats [9]. Finally, researchers also have reported that mice fed high-fat diets to induce hyperlipidemia have reduced failure stress and load-to-failure at the patellar tendon [12].

The damage to the tendons appears to result from multifactorial causes. For instance, the accumulation of cholesterol byproducts impairs blood circulation in the tendon [13, 14]. Moreover, hyperlipidemia also alters broad cellular processes, including biosynthesis of structural macromolecules, formation of supramolecular fibrillar assemblies, and matrix metalloproteinase (MMP)-controlled matrix remodeling [15, 16].

Our study addresses a gap in understanding the relation between hypercholesterolemia and the weakening of tendons. Here, we aim to fill this gap by defining the consequences of excess cholesterol on the integrity of the fibrillar architecture of the Achilles tendon in a rabbit model of hypercholesterolemia. We hypothesize that the infiltration of cholesterol weakens the tendon structure via a mechanism that involves damage to the collagen fibrils due to intimate cholesterol-collagen contact.

## Material and methods

### Animal model

Procedures performed on animals were approved by the Thomas Jefferson University’s Institutional Animal Care and Use Committee. No animals were alive at any point during our study. Specifically, we obtained permission to collect discarded tendons from an unrelated study on the formation of atherosclerotic plaques. Except for causing hypercholesterolemia, procedures associated with the studies on the atherosclerotic plaques did not have any impact on the tendons.

To estimate the number of rabbits needed for our study, we considered published research data on the mechanical characteristics of the tendons from rabbits fed with a high-cholesterol diet similar to that applied here [17]. In this study, the authors determined the differences between the load-to-failure values measured for the supraspinatus tendons in rabbits fed a normal diet or a high-cholesterol diet. Considering the differences between the means, together with the standard deviations, we calculated that we will need 3 rabbits per group to achieve 95% power (with two-sided significance level *α* 0.05) in the similar assays. We determined the sample size using GraphPad StatMate version 2.00 for Windows (GraphPad Software, San Diego, CA)

The Achilles tendons we utilized were obtained from euthanized female rabbits (hCH, *n* = 5) fed 6 oz/day of a high-cholesterol (1%) diet (Research Diets, Inc., New Brunswick, NJ) for 12 weeks. Similar diets containing 0.5 to 1% cholesterol are used routinely in rabbit-based models of hypercholesterolemia [17–19]. This diet was prepared based on the Certified Rabbit Diet 5322 (Purina Mills, Lancaster, PA). Following 12 weeks of a high-cholesterol diet, the rabbits were fed a standard diet for 4 weeks, then sacrificed. Control tendons (nCH, *n* = 5) were obtained from rabbits fed with the same base diet with no cholesterol. The average age of the hCH group was 2.6 years, and the average mass at the time of sacrifice was 3.4 kg. The average age of the nCH group was 2.0 years, and the average mass was 3.5 kg.

### Processing of the tendons

Following sacrifice, the Achilles tendons were harvested; one tendon was preserved for biomechanical tests and the other one, from the contralateral leg, was utilized for preparing tissue sections and collagen extracts. In brief, portions of the mid-substance regions were embedded in optimal cutting temperature compound (OCT, Tissue-Tek), then frozen at − 70 °C. Other portions of the mid-substance regions were fixed in paraformaldehyde and processed for histology. Portions of the tendons flanking the mid-substance were utilized for extraction of collagen.

For the Fourier transform infrared (FTIR) spectroscopy, 3-μm-thick longitudinal sections were prepared from the OTC-embedded samples. These samples were deposited on the MirrIR low-e microscope slides (Kevley Technologies, Chesterland, OH). OTC-embedded sections were also employed for the lipid-specific staining with Oil Red.

### Histology of the tendons

Paraffin-embedded samples from nCH (*n* = 5) and hCH (*n* = 5) rabbits were processed for hematoxylin and eosin staining (H&E) to visualize the general tendon architecture and cellularity. Longitudinal sections were also stained with collagen-specific picrosirius red dye to allow analyses of the organization of the bundles of collagen fibers.

### Collagen hybridizing peptide

We employed a biotinylated form of the collagen hybridizing peptide (CHP; 3-Helix Inc., Salt Lake City, UT) that specifically binds to single α-chains of collagens. Note that CHP does not bind to the α-chains folded into proper triple helices of collagenous proteins. In contrast, CHP binds to free α-chains that do not form proper triple helices due to misfolding or degradation of collagen molecules [20–22].

We applied the biotinylated CHP to the tendon sections from all rabbits, according to the manufacturer’s protocol. The collagen-CHP binding was visualized using a red fluorophore conjugated with avidin. Besides, we stained the nuclei with 4′,6-diamidino-2-phenylindole (DAPI) to visualize the distribution of cells.

### Fourier transform infrared spectroscopy

An FTIR spectrometer (Spotlight 400, Perkin Elmer, Waltman, MA) was used to analyze all tendon samples. For each rabbit, we prepared two tissue samples. Then, on average, we analyzed nine regions of interests (ROI) per sample for the hCH group (total 90 ROIs) and 4.5 ROIs per sample for the nCH group (total 45 ROIs). The reason we selected more ROIs per sample in the hCH group was that the structure of the tendons was not uniform due to the breaks and cell infiltration. In contrast, the structure of the tendons from the nCH group was quite uniform with low cell content.

The tissues were sampled in the trans-reflectance mode using a reflective substrate, MirrIR low-e microscope slides. The measurements were done in the imaging mode in the 4000 to 748 cm^−1^ wavenumber spectral range, at a pixel resolution of 25 μm, with 32 scans per pixel, and a spectral resolution of 8 cm^−1^.

To visualize the distribution and the intensities of the collagen-specific, protein-specific, and lipid-specific average absorbance signals, the following normalized spectral images were generated: (i) at the collagen peak (centered around 1338 cm^−1^; attributed to the CH_2_ wagging vibration of proline side chains), (ii) at the amide II protein peak (centered around 1560 cm^−1^, associated with the N–H bending and C–N stretching vibrations), (iii) at the cholesterol esters peak (centered around 1731 cm^−1^, attributed to the stretching C=O groups), and (iv) at the peak associated with the long unsaturated aliphatic chains of lipids (centered around 2932 cm^−1^, associated with the C–H stretching) [23–25].

Following scanning of the multiple areas of the tendons, co-added spectra were generated with the Spectrum Image software (PerkinElmer, Inc., Waltman, MA). Subsequently, employing the Spectrum software (PerkinElmer, Inc., Waltman, MA), we calculated the ratios of the areas of the integrated amide II and collagen peaks. According to the earlier studies, the increase in this ratio indicates the decrease of the structural integrity of collagen molecules [26–28]. The data points were plotted for each group and demonstrated as the means with the standard deviations (±SD) (GraphPad Software, Inc., La Jolla, CA).

### Analysis of collagen III

We analyzed whether the high-cholesterol diet changes the relative amount of collagen III in the tendons of the hCH rabbits compared to the control nCH rabbits. In brief, we extracted a pepsin-soluble fraction of collagen using porcine pepsin (Sigma-Aldrich, St. Louis, MO) applied at 5 mg/ml in 0.5 M acetic acid. Next, the α1(III) chains were separated from the α1(I) chains using delayed reduction gel electrophoresis, as described [29, 30].

Corresponding samples were applied to a separate gel in which electrophoresis was carried out in reducing conditions applied from the beginning of the electrophoretic run. Following electrophoresis, collagen bands were visualized by staining with Coomassie blue dye.

### Biomechanical tests of tendons

After euthanasia, the Achilles tendon was harvested and immediately frozen in PBS-soaked gauze at − 80 °C until use. No tendons were purposely excluded from the biomechanical tests, but ultimately, four from the nCH group and three from the hCH group were of sufficient size and quality for testing.

Before mechanical testing, each tendon was incubated overnight at 4 °C in PBS. Mechanical testing was performed using an ElectroForce-3200 material testing system (TA Instruments, New Castle, DE). A region of approximately 10 mm at each end of the specimen was clamped securely using thin film grips (model Imada-FC-20, Imada, Inc., Northbrook, IL) attached to a 225-N load cell. First, a preload of 0.3 N was placed on the specimen. The cross-sectional area and gauge length were carefully measured using digital calipers. Next, 10 cycles of preconditioning were performed using a sinusoidal waveform of 5% displacement at 0.2%/s. Finally, a monotonic displacement ramp of 0.1 mm/s was applied until failure. Force and displacement were acquired at 25 Hz and analyzed digitally using a custom GNU/Octave script. The data points were plotted for each group and demonstrated as the means with the standard deviations.

### Atomic force microscopy-based nanoindentation

Each OCT-embedded tissue was cryo-sectioned longitudinally along the fiber axis onto the glass slides to produce ~ 5-μm-thick sections. Following cryopreservation, the samples were stored in − 80 °C in OCT for less than 1 week until AFM nanoindentation tests. Only intact OCT-frozen sections were selected for the AFM assays. Sections missing any fragment were excluded. Consequently, selected tendons from the nCH (*n* = 3) and the hCH rabbits (*n* = 3) were processed for the AFM indentation studies.

For each rabbit, we prepared two tissue sections. Prior to testing, the sections were thawed and washed in phosphate-buffered saline (PBS). On each section, AFM nanoindentation was performed using microspherical colloidal tips (*R* ~ 5 μm, nominal *k* = 0.6 N/m, HQ:NSC36/Tipless/Cr-Au, cantilever C, NanoAndMore, Watsonville, CA) and a Dimension Icon AFM (BrukerNano, Santa Barbara, CA) at 10 μm/s indentation rate up to a maximum load of ~ 120 nN [31]. Given the tip radius ~ 5 μm and a maximum indentation depth ~ 0.5–1 μm, the effective tip-sample contact radius was ~ 2–3 μm. At this scale, the indentation modulus is a manifestation of local sliding and uncrimping of the collagen fibrils and, thus, is highly sensitive to the structural integrity of tendon.

For each sample, we performed nanoindentation at three random sites. At the same location, nanoindentation was repeated at least three times, where the high repeatability of indentation curves suggested negligible irreversible plastic deformation. At each location, the effective indentation modulus, *E*_ind_ (in Pa) was calculated by fitting the entire portion of each loading force-depth curve to the finite thickness-corrected Hertz model via least squares linear regression by assuming Poisson’s ratio, *ν* = 0.3 for tendon [32, 33].

### Statistical plan

We derived the number of animals we needed for our study based on the work published by Chung et al. as described above [17]. We employed a one-way analysis of variance (ANOVA; IBM SPSS Statistics, v25) to determine whether there are any statistically significant differences between the means calculated for the hCH and the nCH groups (significance level 0.05). For all analyzed parameters we measured, the data points were plotted for each group and presented together with the means and the standard deviations. The significance levels were indicated with asterisks in all graphs: *P < 0.05, **P < 0.01; ***P < 0.001

## Results

### Animal model

Biochemical assays demonstrated the increase of cholesterol in the blood of the hCH rabbits. While the concentration of total cholesterol in the nCH rabbits ranged from 10 to 80 mg/dl, the concentration of total cholesterol in the hCH rabbits ranged from 900 to 1200 mg/dl. No adverse effects of the high-cholesterol diet were observed in the hCH group. Both groups exhibited the same level of daily activities, and their masses were comparable.

### Histology of tendons

**Fig. 1:**
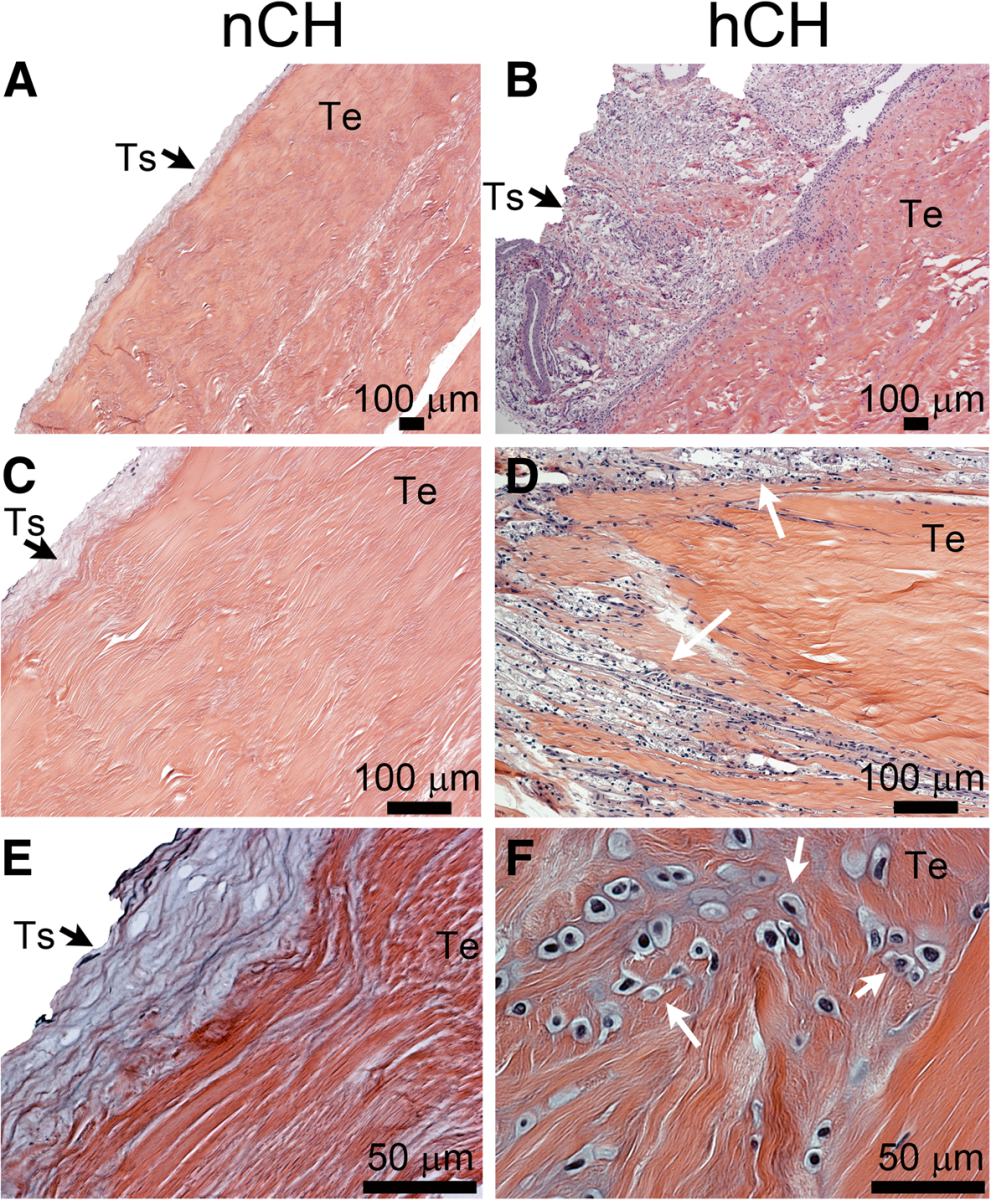
A general histology of the Achilles tendons from the nCH (**a**, **c**, **e**) and the hCH (**b**, **d**, **f**) rabbits. Individual panels stained by H&E show increasing magnifications of the tendon fragments. Arrows seen in **d** and **f** indicate cell infiltration. Ts, tenosynovium; Te, tendon

**Fig. 2:**
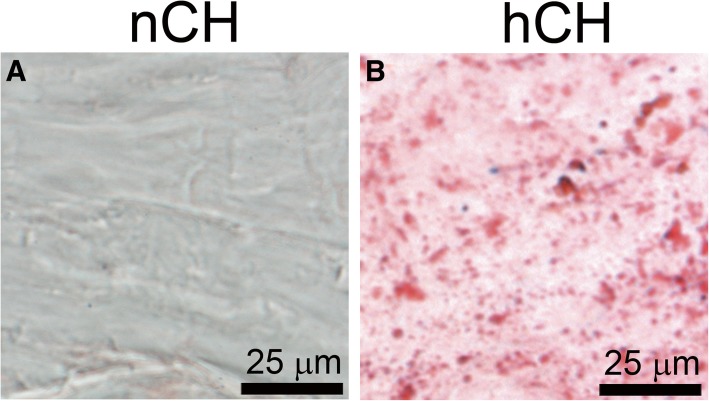
Oil Red staining of lipid deposits in tendon samples from the nCH rabbits (**a**) and hCH rabbits (**b**). The deposits seen in **b** most likely include esterified cholesterol

Following isolation, the Achilles tendons were processed for the H&E staining and then observed under a microscope (Fig. 1). These observations revealed marked differences in the nCH and the hCH tendons. First, the cellularity of the nCH tendons was low with only a few tenocytes visible in the tendon sections. In contrast, the hCH tendons were characterized by hypercellularity with cells, including numerous clusters with morphology similar to macrophages, infiltrating the tendon structure (Fig. 1f). The tenosynovium also was hypercellular with marked hyperplasia. Meanwhile, the thickness of tenosynovium of the nCH tendon was uniform, and the content of cells was relatively low. Finally, the Oil Red staining revealed lipid-rich deposits in the tendons from the hCH rabbits while, in contrast, similar deposits were not present in the nCH tendons (Fig. 2).

### Hybridization of tendon tissue with CHP

**Fig. 3:**
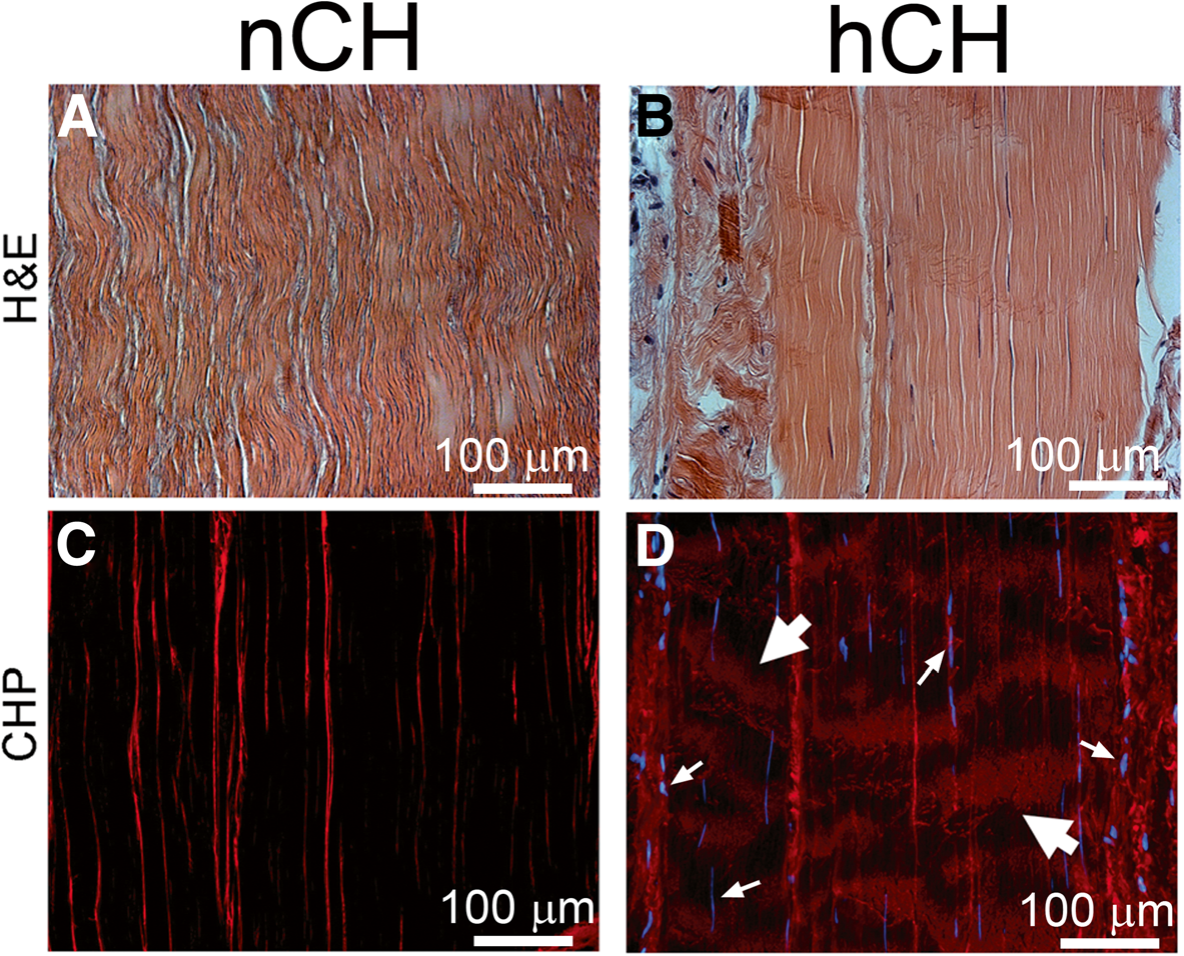
Detection of CHP-accessible collagen α-chains. **a**, **b** H&E staining of the tendons from the nCH and the hCH rabbits. In addition, **c** and **d** demonstrate the CHP-positive signals in the consecutive sections observed using a fluorescence microscope. While the CHP-positive staining in the nCH samples is seen exclusively at the edges of the bundles of collagen fibers that form the tendon architecture, in the hCH samples, this staining is also seen across (large arrows) the longitudinal sections of the analyzed tendons. Besides, the DAPI staining corroborates a marked increase in the cellularity (small arrows) of the HCH tendons (**d**) in comparison with control (**c**)

**Fig. 4:**
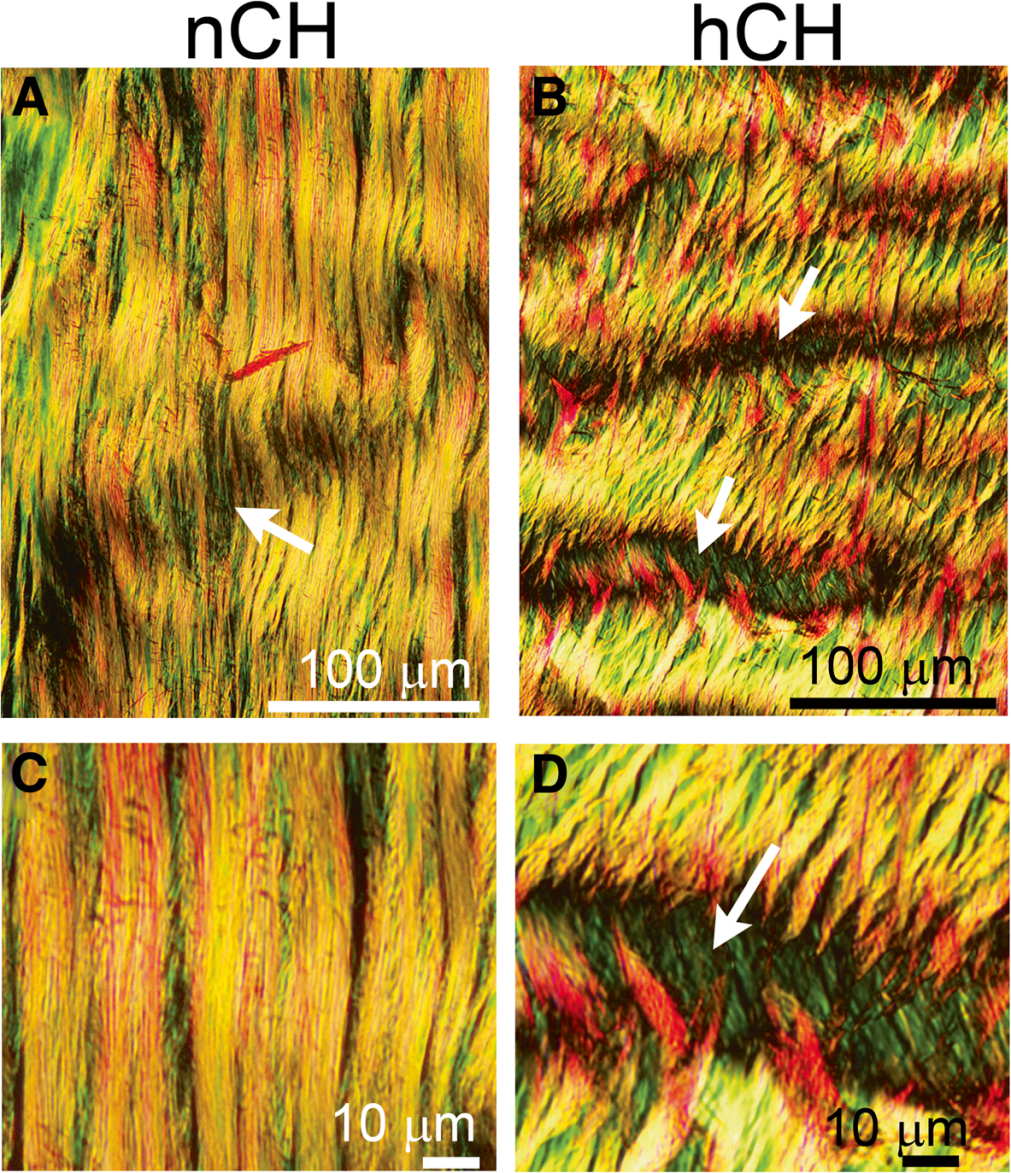
The architecture of the tendons visualized by staining with picrosirius red. While **a** and **b** show a relatively low magnification of the tendons, **c** and **d** show relatively large magnifications of the selected regions. Although the bundles of collagen fibers in the nCH rabbits (**a**, **c**) are uninterrupted, the bundles of collagen fibers in the hCH rabbits (**b**, **d**) have readily visible breaks (arrows). The arrow in **a** shows a crimped area along the collagen fibers. Unlike the breaks observed in **b** and **d**, the indicated crimp area remains intact

While intact collagen α-chains fold into tightly packed triple helices, misfolded or cleaved α-chains unfold, thereby allowing their binding with the CHP. Applying the CHP to the tendons from the nCH and hCH rabbits, we showed the binding of this peptide to the collagen-rich structure of the tendons (Fig. 3), and we observed marked differences between the binding patterns. Specifically, in the nCH tendons, the CHP-positive signals were only observed along the edges of the collagen fibers parallel to the longitudinal axis of the tendon, while in the hCH tendons, the CHP-specific signals were also observed in the form of the bands running perpendicular to the long axis (Fig. 3c, d). This unique pattern of hybridization of the CHP most likely indicated the sites of the unfolding of collagen triple helices at points of the α-chains degradation. Observation of the breaking of collagen fibers in the hCH tendons stained with collagen-specific picrosirius red supports this same pattern of binding (Fig. 4). Specifically, in contrast to control (Fig. 4a, c), the collagen fiber continuity in the hCH tendons was interrupted (Fig. 4b, d). Moreover, observation of the picrosirius red-stained samples did not reveal any readily visible signs of fibrosis.

### FTIR assays of tendons

**Fig. 5:**
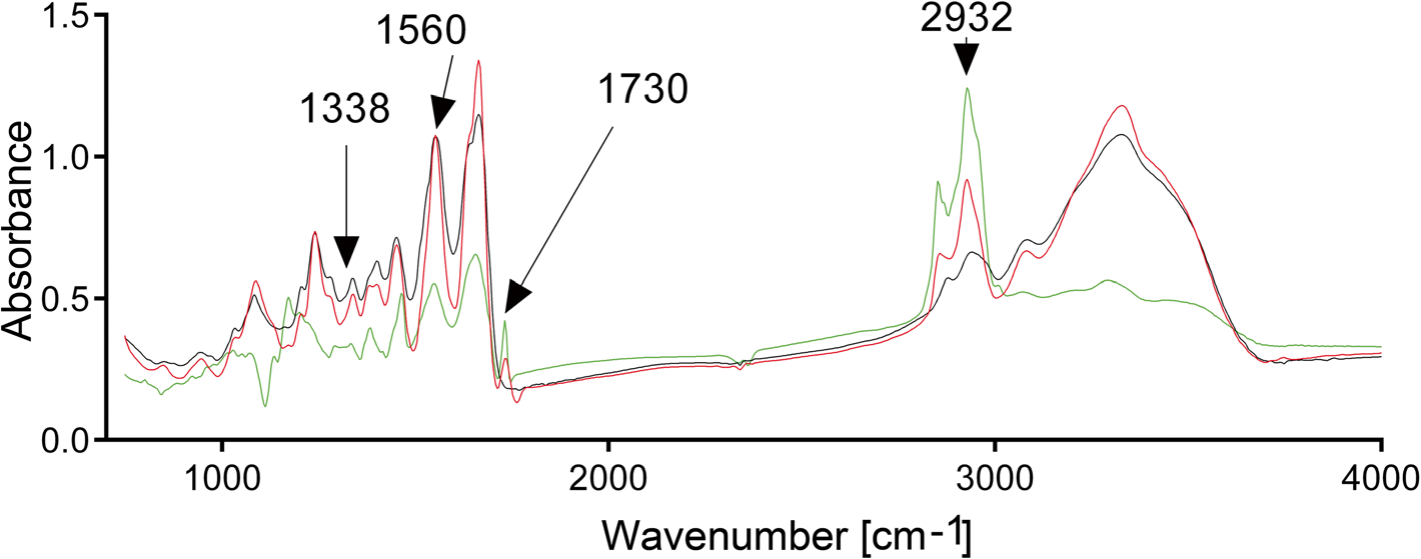
Representative FTIR spectra of the tendon sections derived from the nCH rabbits (black line) and the hCH rabbits (red and green lines). Principal peaks evaluated in this study are demonstrated. The peak of absorbance corresponding to the collagen region centers around 1338 cm^−1^ and the peak corresponding to the protein region centers around 1560 cm^−1^. Distinct peaks at 1730 cm^−1^ and 2932 cm^−1^ are associated with cholesterol esters

Due to the unique physicochemical properties of analyzed molecules, we were able to obtain protein-specific, collagen triple helix-specific, and lipid-specific signals using the FTIR spectroscopy. Utilizing these specific signals, we analyzed the relative amounts of total proteins, collagen, and lipids. Analysis of the tendon sections from the hCH rabbits revealed the presence of distinct peaks at 1730 cm^−1^ and 2932 cm^−1^ (Fig. 5). Prior studies on non-calcified atherosclerotic plaques demonstrated that these peaks are associated with cholesterol esters [23, 34, 35].

**Fig. 6:**
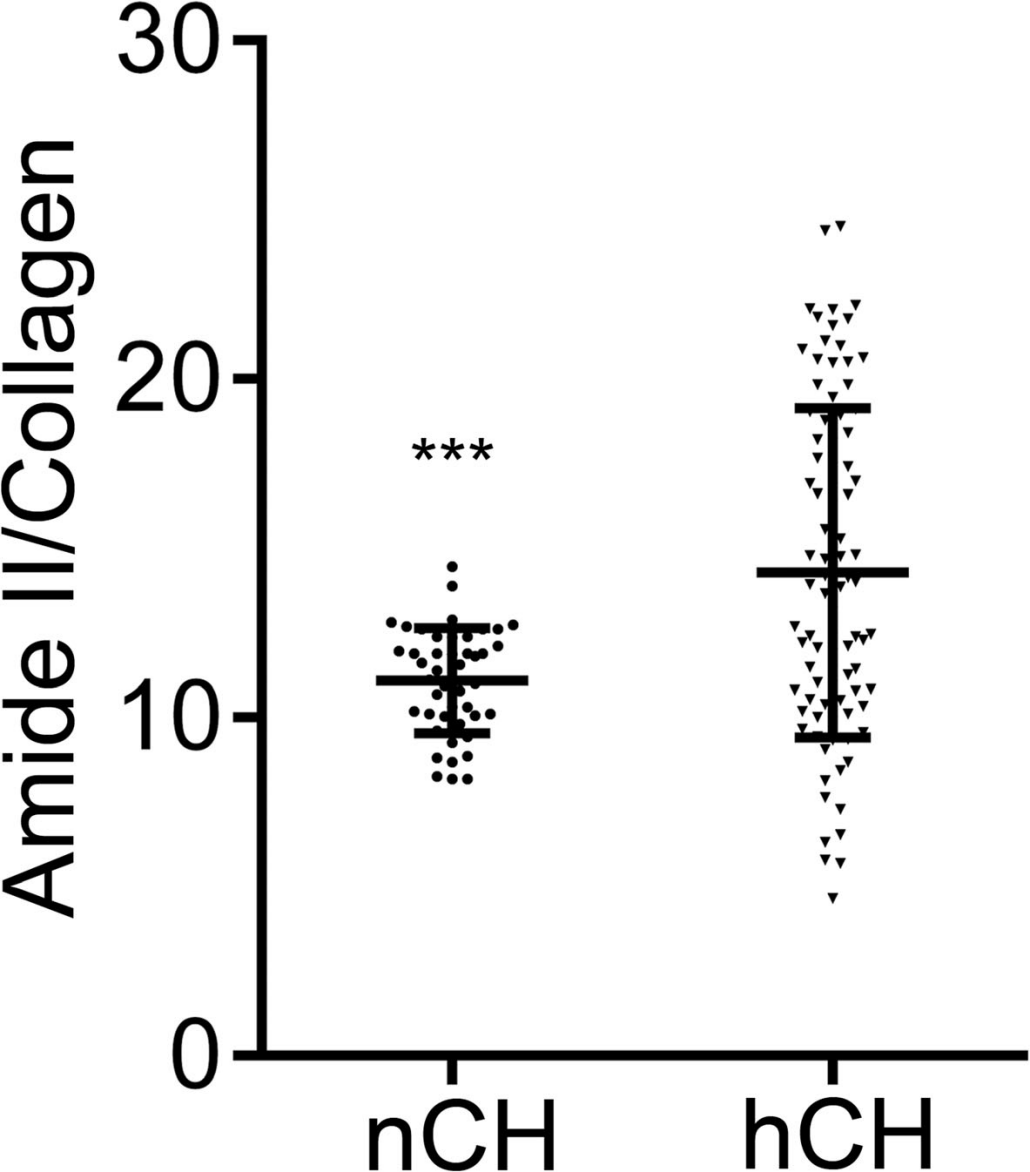
Amide II/1338 cm^−1^ peak area ratio comparison (***P < 0.001). The means ± SD are indicated

**Fig. 7:**
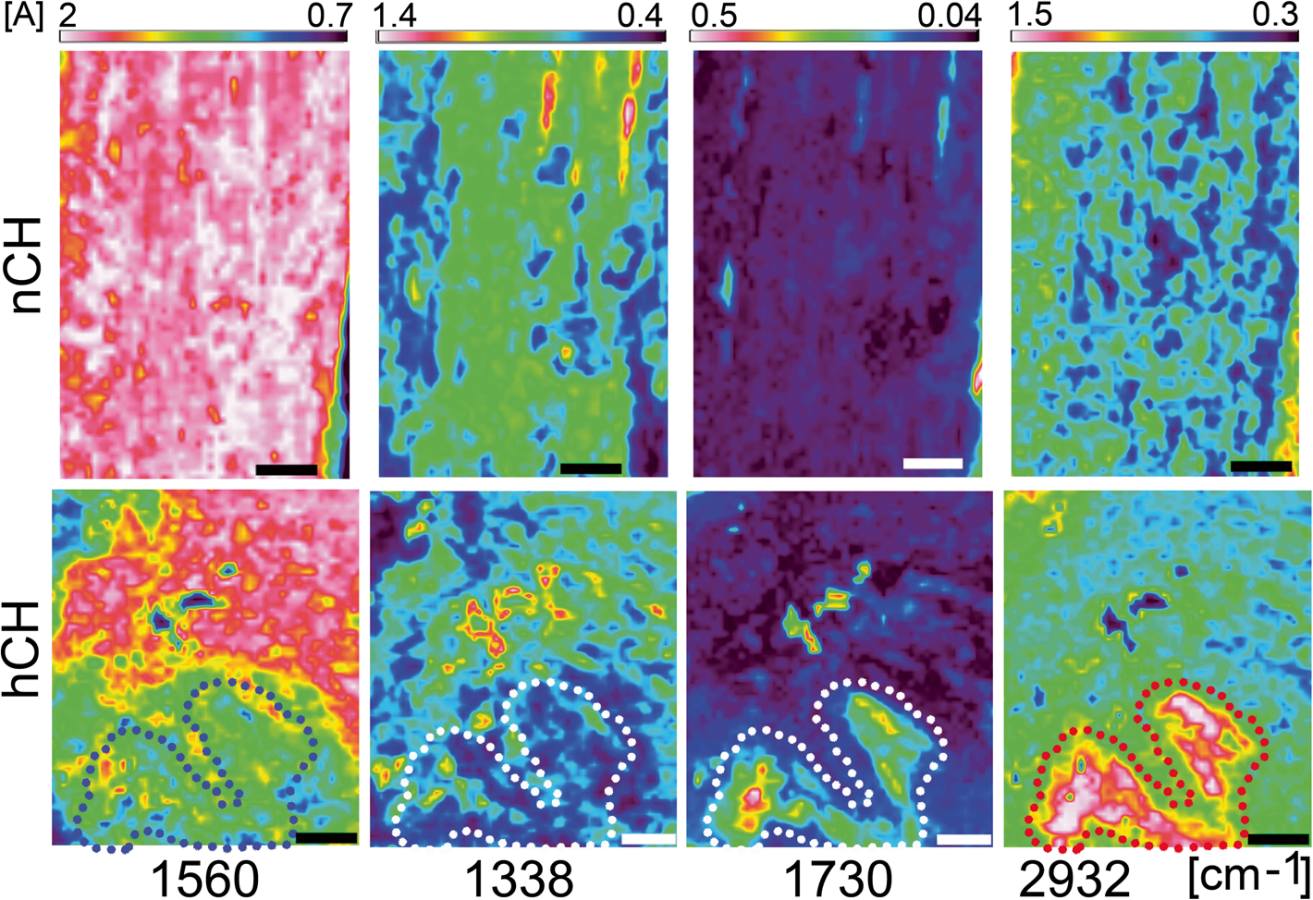
FTIR intensity images of the tendons created by peak integration mapping of the 1338 cm^−1^, 1560 cm^−1^, 1730 cm^−1^, and 2932 cm^−1^ absorbance bands. Tendons from the nCH rabbits have a uniform distribution of protein-specific and the collagen-specific signals. The intensity of the lipid-specific signals in these tendons is relatively low. In tendons from the hCH rabbits, the distribution of protein-specific and collagen-specific signals is not uniform. In these tendons, the areas of lipid-specific signals are evident (delineated with dotted lines). Of note is the observation that in the lipid-rich areas, the intensity of protein-specific and collagen-specific signals is relatively low. Intensity scales [A] are placed on the top. Bars = 200 μm

We also analyzed the ratios of integrated peak areas of the absorbance in the protein region centering around 1560 cm^−1^ and the collagen region centering around 1338 cm^−1^. Measurements of various regions of the tendons from the hCH rabbits demonstrated that this ratio trended significantly higher in comparison with control (Fig. 6). According to Kim et al., this change may signify a decrease of the structural integrity of collagen triple helices [26]. Of interest, we observed that the content of the collagen-derived signal was relatively low in the areas in which the lipid-rich signals were relatively strong (Fig. 7).

### Analyses of collagen III

**Fig. 8:**
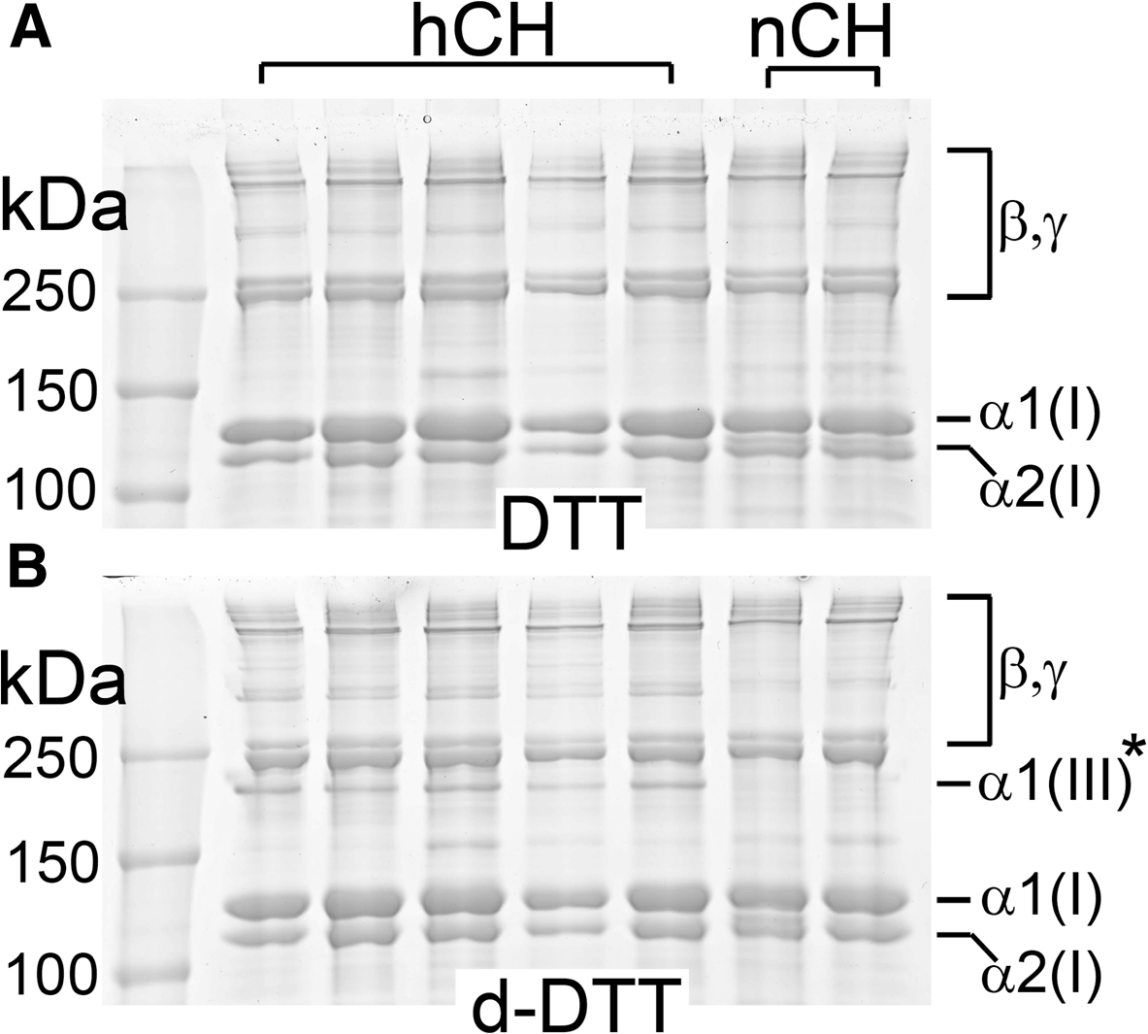
Electrophoresis of collagens extracted from the tendons dissected from the nCH and the hCH rabbits. Patterns of migration of collagen I and collagen III chains separated in standard (**a**) reducing conditions (DTT) and in delayed reduction (B) conditions (d-DTT) of electrophoresis. α1(I), α2(I): specific chains of collagen I; α1(III)*: collagen III chains separated with the use of delayed-reduction conditions; β, γ: oligomers consisting of cross-linked collagen α-chains. Molecular mass markers (kDa) are also indicated

As the composition of the fibrillar collagen types may change due to many pathological processes, we analyzed the relative content of two major collagen types that build the architecture of the tendon, namely collagen I and collagen III. Employing a method that allows separation of the α-chains of these collagens, we demonstrated an increase in the collagen III to collagen I ratio in the hCH rabbits; there was an about fourfold increase in this ratio, from 0.14 in the nCH rabbits to 0.6 in the hCH rabbits (Fig. 8).

### Mechanical tests

**Fig. 9:**
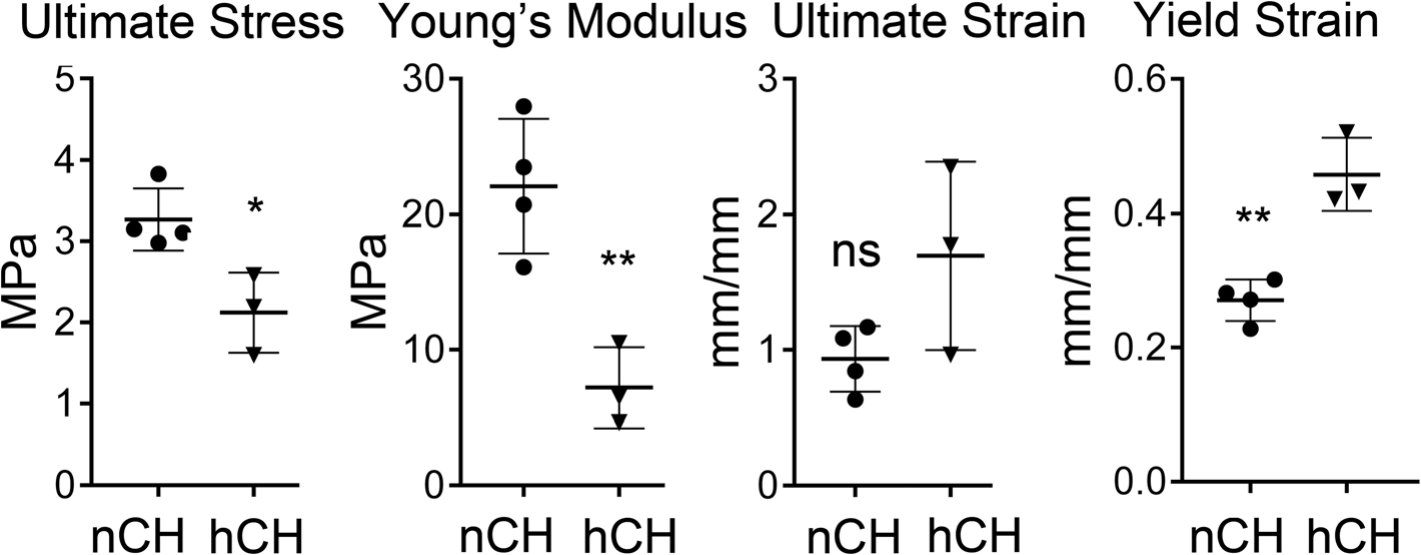
A graphic representation of mechanical properties of the nCH and hCH tendons. The means ± SD are indicated. The statistical significance of the differences between pairs of the means are indicated (*P < 0.05; **P < 0.01; ***P < 0.001; ns, no statistical difference)

To analyze the mechanical properties of the tendons from the hCH (*n* = 4) and nCH (*n* = 3) rabbits, we performed monotonic tensile tests to failure on the Achilles tendon samples (Fig. 9). Our results demonstrate that the high-cholesterol diet is associated with diminished tendon strength and stiffness of the hCH rabbits, as illustrated by marked decreases in the ultimate stress and Young’s modulus. Moreover, we observed a higher yield strain in the high-cholesterol tendons. In total, our results indicate that high cholesterol markedly impairs tendon biomechanical performance, as characterized by diminished strength and stiffness with increased laxity.

### Nanoindentation

**Fig. 10:**
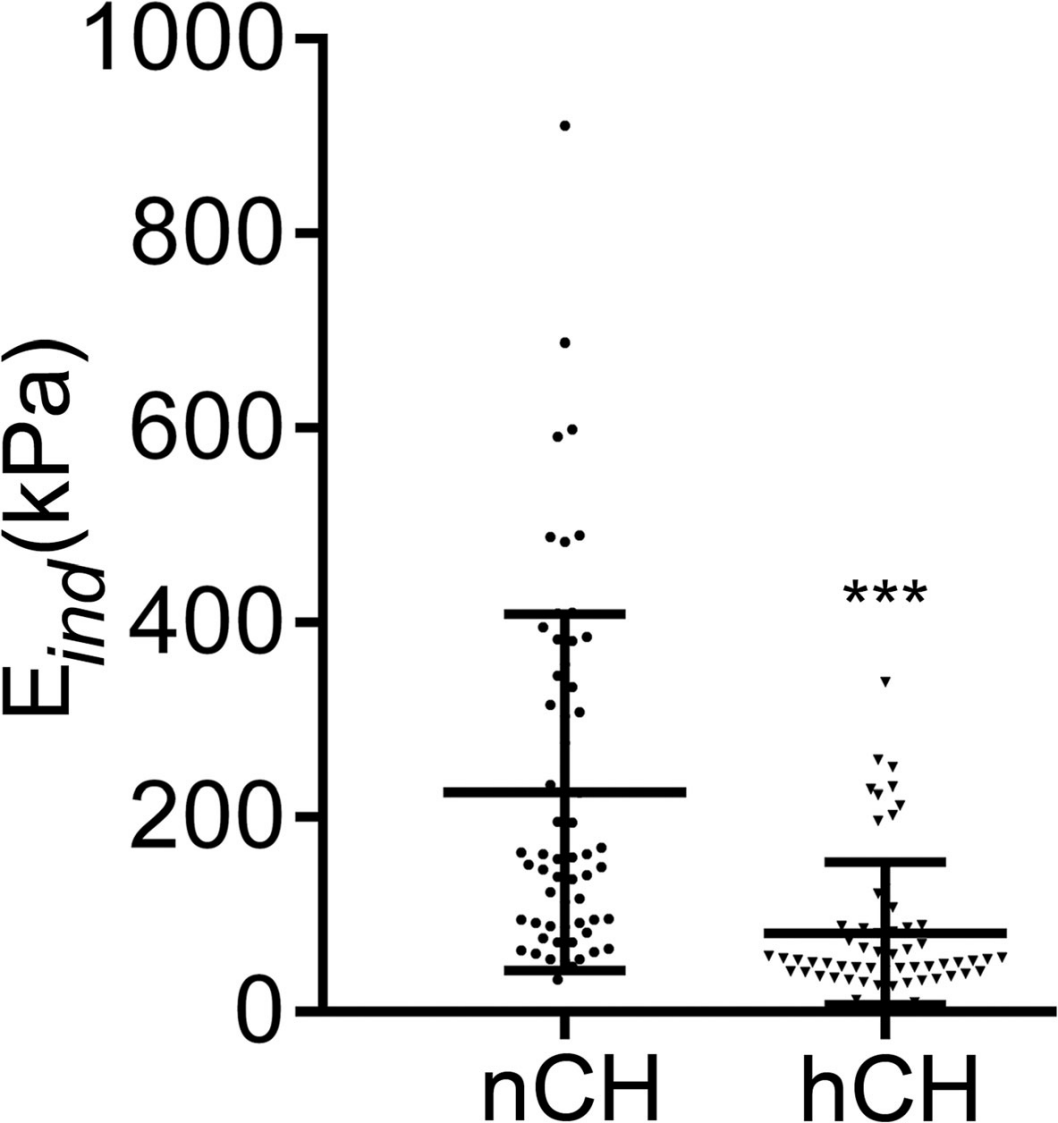
A graphic representation of nanomechanical properties of the nCH and hCH tendons. The means ± SD are indicated. The statistical significance of the difference between the means are indicated (***P < 0.001)

We performed nanoindentation assays on randomly selected sections from the hCH and the nCH rabbits. AFM nanoindentation detected a remarkably smaller indentation modulus for the hCH tendons compared to the nCH control (Fig. 10).

## Discussion

Poor mechanical characteristics and alteration of the healing of the tendons due to hyperlipidemia present a serious clinical problem. Scientists have hypothesized tendons exposed to high concentrations of lipids develop molecular-level damage to the collagenous architecture that leads to injury and impairs healing [8, 36]. Based on the similarities to pathological processes taking place in atherosclerotic plaques, researchers have studied the potential mechanisms of tendon damage. These mechanisms include (i) reduced expression of genes encoding collagen I, (ii) increased expression of MMPs, (iii) reduced expression of tissue inhibitors of MMPs (TIMPs), (iv) intracellular accumulation of toxic cholesterol deposits that trigger deleterious chronic inflammation and may activate mitochondrial pathways of apoptosis, and (v) direct binding of cholesterol to the collagen fibrils that alters their arrangement [1, 12, 37–43]. Despite these studies, the effects of hyperlipidemia on tendon damage have remained ill-defined.

The findings from our study support the hypothesis that the high cholesterol diet leads to the weakening of the tendons via a mechanism that involves damage to the collagen fibrils. Detecting the exposed collagen α-chains within the tendons’ architecture validates this hypothesis. We propose that these single α-chains occurred because collagen molecules were damaged. Although our study could not answer whether this damage resulted due to enzymatic or physical cleavage of collagen molecules, we postulate that it was site-specific. In support of this notion, we observed a remarkably regular pattern of breaks in the fibrils that form the architecture of the tendon.

We predict that when the collagen molecules in the fibrils break, the collagen then becomes degraded. Degradation of collagen molecules occurs because when the individual collagen α-chains that form the triple helices break into smaller fragments, their thermostability drops below the body temperature [44, 45]. This drop in the thermostability prompts the fragments to unfold into individual chains, thereby rendering them a target of proteolytic enzymes. Although very slow, the above process also takes place in physiological conditions to allow the normal turnover of collagen fibrils. The physiological degradation of collagen molecules depends on the catalytic activities of MMPs, most notably MMP-1. MMP-1 is an enzyme that cleaves the fibrillar collagens at a defined site into the two thirds and the one third fragments that, unlike the original full-length parent molecule, unfold below 37 °C [45].

As indicated by our FTIR analysis and calculations of the amide II/collagen ratios, the areas of relatively low collagen content in the Achilles tendons from the hCH rabbits overlap with the regions of the relatively high cholesterol content. Consistent with the studies on the degradation of the collagen matrix in the cartilage, our observation of the decrease of the intensity of collagen-specific signals may indicate reduced integrity of collagen molecules [28]. This overlap of the regions of collagen disintegration with the regions of high-lipid content may indicate an excessive accumulation of the lipids within the collagen fibrils present in those areas. Scientists well recognize a strong interaction of extracellular lipids with collagen fibrils. For instance, in human xanthomas, like in atherosclerotic plaques, unesterified cholesterol accumulates mainly in the extracellular space while Oil Red-stained esterified cholesterol accumulates both intracellularly and extracellularly [40]. Notably, Rabinowitz and Shapiro have demonstrated that collagen molecules have a strong propensity to interact with lipids, with a significant portion of lipids remaining bound to collagen isolated from the skin even after the treatment with organic solvents [46].

Considering these characteristics of the lipid-collagen binding interaction, we suggest that this binding could be a part of a mechanism that damages the collagen fibrils and thereby alters their mechanical properties. In this context, we agree with earlier suggestions that the lipid-associated damage to the tendons could result from chronic processes causing recurrent microdehiscence of the tendon tissue [36]. Specific mechanisms of the fibril damage, however, remain unclear.

Mechanical testing of the tendons showed diminished tendon strength and stiffness. The absolute values from our biomechanical testing are somewhat lower than expected for this tissue type, which may be due to the possible differences in tissue processing, measurements of tendon cross-sectional area, or gauge length measurements. Also, our data differ from those of Beason et al. who demonstrated increased stiffness of the supraspinatus tendon in hypercholesterolemic mice, rats, and monkeys [11]. Still, the same group reported normal or decreased stiffness of the patellar tendons in hypercholesterolemic mice [8]. Moreover, Grewal et al. reported that mice fed a high-fat diet had reduced patellar tendon failure stress [12]. Here, our data indicate that hypercholesterolemia negatively affects the biomechanical performance. However, in the context of the studies referenced above, this finding may depend on the age of the animal, the type of tendon, and specific biomechanical test performed.

The nanoindentation studies further support our observation of diminished mechanical stability of tendons from the hCH rabbits. Utilizing a design with an effective tip-sample contact radius of about 3 μm, we analyzed a relatively large region of a collagen fibril. At this scale, the indentation modulus is a manifestation of local sliding and uncrimping of the collagen fibrils rather than mechanical properties of the gap zone, within which, as described above, collagen fibril-lipid binding takes place [47]. Considering that the length of a gap-overlap region is 67 nm, the tip we employed covered a number of the gap-overlap domains. Thus, the method we used is highly sensitive to detect the structural integrity of the fibrillar architecture of tendon rather than the measure properties at the ultrastructural level. AFM data are consistent with our hypothesis that high cholesterol results in impaired collagen fibril structure in the tendon at the microscale.

Due to the similarities between the processes occurring in hyperlipidemia in the atherosclerotic plaques and tendons, one possible mechanism for the collagen fibril damage could involve persistent inflammation and increased production of macrophage-derived MMPs. This notion is supported by the fact that, similar to the results presented here, researchers have demonstrated the presence of lipid-loaded macrophages in earlier studies on tendon xanthomas [36].

Furthermore, hyperlipidemia also alters broad cellular processes, including biosynthesis of collagenous and non-collagenous structural macromolecules and matrix remodeling. For example, studies of the atherosclerotic blood vessels and xanthomas have demonstrated significant qualitative and quantitative changes in collagen I and collagen III and increased proteolytic activity of specific MMPs [15, 16]. We further illustrated these changes by a remarkably increased collagen III to collagen I ratio in the hCH tendons. As the increase of the collagen III to collagen I ratio is frequently reported in fibrotic tissues, we cannot exclude a possibility that prolonged inflammation due to hypercholesterolemia triggered the fibrotic response. We contemplate, however, that this increase of the collagen III to collagen I ratio might be hypercholesterolemia-specific. In particular, when studying post-traumatic arthrofibrosis in a rabbit model of the knee injury, we also analyzed the collagen III to collagen I ratio in fibrotic posterior knee capsules [30]. Although we observed a slight increase in the ratio, this increase was minimal. Moreover, while we observed an excessive accumulation of collagen-rich deposits in the fibrotic joint capsules, here, similar deposits within the Achilles tendons of the hCH rabbits were not evident.

Another intriguing possibility for lipid-dependent damage to the collagen fibrils was presented by Chapman and colleagues who studied the interactions of the collagen fibrils in vitro with non-polar liquids. They concluded that collagen fibril/non-polar compounds interaction is site-specific and that the binding occurs in the gap region of the collagen fibrils [47]. The authors demonstrated that binding non-polar compounds breaks the fibrils over considerable regions. They also suggested that infiltration of cholesterol into the collagen fibrils in the atherosclerotic plaques, in the arcus juvenilis of the cornea, and infiltration of lipids to the dermis in diabetes mellitus may reduce the mechanical strength of collagenous framework of these tissues by the same mechanism.

Moreover, the authors have shown that these breaks occur within individual collagen molecules that build a fibril. The authors explained that splitting the collagen molecules results from the binding of non-polar agents to a unique hydrophobic region present within the collagen fibrils. This unique region consists of clusters of hydrophobic amino acid residues whose presence defines a highly flexible domain of collagen triple helix. The authors presented evidence that binding non-polar agents to this flexible region stiffens it. This stiffening could weaken the fibril by reducing the mobility of the lipid-binding region. A similar reduction of the mobility of triple helical domains was observed with gelatin absorbed onto hydrophobic surfaces in an oil-water emulsion [48]. The breakage occurs in the proximity to the fibril-stabilizing cross-links, thus further altering the structural integrity of a collagen fibril. Our findings align with research by Chapman et al. This alignment is evident by the presence of collagen fibril breaks and the overlap of lipid-rich regions with the regions of decreased collagen content seen in the tendons from the hCH rabbits.

Moreover, studies demonstrated that binding non-polar compounds to free collagen molecules reduces their ability to incorporate into a fibril [47]. If correct, this could explain the alterations of healing of injured tendons and ligaments seen in hyperlipidemia [9]. The specific pathomechanism of these alterations is that binding of lipids to free collagen molecules blocks their self-assembly into the collagen fibrils needed to repair the architecture of damaged tissue. A similar mechanism of blocking collagen fibrillogenesis by binding of small molecules and antibodies to various sites of free collagen molecules was reported by various researcher groups [49, 50].

Chapman et al. also contemplated that binding of non-polar compounds to the hydrophobic domain of collagen molecules may displace the water shell surrounding the α-chains, thereby altering the physicochemical characteristics of the triple helices [47]. Also, the same authors discuss their observation on a substantial reduction of the wet tensile strength and stiffness of the rat tail tendons treated with hydrophobic compounds [47]. Collectively, these data support our observation of the tendon weakening in the hCH group.

The main limitation of this study in the context of human disease is that the concentrations of cholesterol in the sera of analyzed rabbits were extremely high relative to those observed in typical hypercholesterolemic patients. Thus, we cannot exclude the possibility that the changes that we observed in the tendons were more dynamic than the changes in humans with chronic hypercholesterolemia. Another limitation is a relatively small number of animals available for this study. Thus, we consider our work introductory and contemplate further studies with more comprehensive experimental design.

## Conclusions

Despite the limitations, our study describes the molecular-level consequences of infiltration of cholesterol into the collagen-rich architecture of the tendon. According to our knowledge, this study offers the first FTIR-based and CHP-based evidence for the colocalization of cholesterol deposits with damaged collagen fibrils. This new indication supports the hypothesis that intimate cholesterol-collagen contact may be a critical factor in damage of the collagen-rich framework of the tendons associated with hypercholesterolemia.

## Data Availability

All data generated or analyzed during this study are included in this published article.

## Abbreviations

AFM: Atomic force microscopy
CHP: Collagen hybridizing peptide
DAPI: 4′,6-Diamidino-2-phenylindole
FTIR: Fourier transform infrared spectroscopy
H&E: Hematoxylin and eosin
hCH: High cholesterol
LDL: Low-density lipoprotein
MMP: Matrix metalloproteinase
nCH: Normal cholesterol
PBS: Phosphate-buffered saline
ROI: Region of interest
SD: Standard deviation
TC: Total cholesterol
TG: Triglycerides
TIMP: Tissue inhibitor of matrix metalloproteinases
